# Interaction Between *DRD2* rs1076560 Genotype and Stimulant Dependence on Impulsivity and Self-Reported ADHD Traits in Men

**DOI:** 10.3390/neurolint17110182

**Published:** 2025-11-05

**Authors:** Milena Lachowicz, Remigiusz Recław, Jolanta Chmielowiec, Krzysztof Chmielowiec, Kinga Łosińska, Dariusz Larysz, Anna Grzywacz

**Affiliations:** 1 Department and Clinic of Oncology and Radiotherapy, Medical University of Gdansk, ul. M. Skłodowskiej-Curie 3a, 80-210 Gdansk, Poland; milena.lachowicz@awf.gda.pl; 2 Department of Psychology, Gdansk University of Physical Education and Sport, Kazimierza Górskiego 1 St., 80-336 Gdansk, Poland; 3 Department of Medical Sciences and Public Health, Gdansk University of Physical Education and Sport, Kazimierza Górskiego 1 St., 80-336 Gdansk, Poland; remigiusz.reclaw@awf.gda.pl (R.R.); kinga.losinska@awf.gda.pl (K.Ł.); 4 Department of Nursing, Collegium Medicum, University of Zielona Góra, 28 Zyty St., 65-046 Zielona Góra, Poland; 5 Department of Hygiene and Epidemiology, Collegium Medicum, University of Zielona Góra, 28 Zyty St., 65-046 Zielona Góra, Poland; chmiele@vp.pl; 6 109th Military Hospital with Polyclinic, Ministry of National Defense, ul. Ksiedza Piotra Skargi 9/11, 71-422 Szczecin, Poland; dariuszlarysz@hotmail.com

**Keywords:** *DRD2* rs1076560, stimulant dependence, impulsivity, ADHD traits, BIS-11, dopamine receptor, genetic polymorphism, gene × environment interaction

## Abstract

Background and Objectives: The dopamine D2 receptor (*DRD2*) plays a central role in fronto-striatal circuits regulating cognitive control and reward processing. The rs1076560 polymorphism alters receptor isoform expression, potentially modifying impulsivity and vulnerability to stimulant use disorders. We examined gene–environment interactions between rs1076560 and stimulant dependence in relation to impulsivity, ADHD traits, and hedonic capacity. Methods: A total of 517 men (235 stimulant-dependent, 282 controls) completed the Barratt Impulsiveness Scale (BIS-11), Adult ADHD Self-Report Scale (ASRS v1.1), and Snaith–Hamilton Pleasure Scale (SHAPS). Genotyping for rs1076560 was performed using real-time PCR, and two-way ANOVAs tested genotype-by-group effects. Results: Significant genotype-by-group interactions were observed across all BIS-11 subscales and ASRS scores. In the stimulant-dependent group, C/C homozygotes showed the highest levels of attentional impulsivity and attentional dysregulation compared to both A/C and C/C controls. In contrast, within the control group, A/A homozygotes demonstrated higher motor impulsivity, non-planning impulsivity, and BIS-11 total scores than C/C controls. No significant main effects or interactions were found for SHAPS scores. Conclusions: *DRD2* rs1076560 moderates impulsivity-related traits through dopaminergic pathways relevant to executive dysfunction in stimulant use disorders. These findings highlight a neurobiological mechanism of addiction vulnerability and may inform precision approaches in neurology and psychiatry.

## 1. Introduction

The dopaminergic system plays a central role in regulating reward processing, cognitive control, and motivational salience [1,2]. Dopamine D2 receptors, encoded by the *DRD2* gene, are particularly important for modulating synaptic dopamine availability within mesolimbic and mesocortical circuits [3]. Reduced D2 receptor availability in striatal and prefrontal regions has been linked to diminished inhibitory control, heightened reward sensitivity, and increased vulnerability to maladaptive behaviors [4].

One functional single nucleotide polymorphism (SNP) within *DRD2* that has received growing attention is rs1076560 [5]. This intronic variant alters pre-mRNA splicing, shifting the balance between the short presynaptic (D2S) and long postsynaptic (D2L) isoforms of the receptor. The A allele has been associated with decreased expression of D2S, potentially modifying dopaminergic tone in brain regions implicated in executive functioning and reward regulation [6]. Prior studies have linked rs1076560 to differences in neural activation during cognitive control tasks, susceptibility to addictive behaviors, and variability in personality traits such as risk-taking and decision-making [7,8,9,10,11]. However, evidence remains inconsistent, and relatively few studies have examined its role in the context of stimulant use disorders.

Impulsivity—a multifaceted psychological construct—represents a key behavioral dimension relevant to *DRD2* function and stimulant addiction [12]. It encompasses attentional impulsivity (difficulties sustaining focus), motor impulsivity (acting without forethought), and non-planning impulsivity (lack of future-oriented thinking) [13]. High levels of trait impulsivity are strongly associated with risky decision-making, poor delay of gratification, and increased sensitivity to the reinforcing effects of stimulant drugs [14,15]. Understanding how genetic variation in *DRD2* interacts with impulsivity-related traits may provide insight into individual vulnerability to stimulant dependence.

Furthermore, while most research has focused on diagnostic outcomes, less is known about the dimensional expression of related traits such as attentional control and hedonic capacity. In this context, self-report tools such as the Barratt Impulsiveness Scale (BIS-11) [16], the Adult ADHD Self-Report Scale (ASRS v1.1, 18-item version developed by the World Health Organization) [17], and the Snaith–Hamilton Pleasure Scale (SHAPS) provide a robust means to assess distinct aspects of behavioral regulation and reward processing across populations [18]. Importantly, the ASRS was employed in the present study not as a diagnostic tool for ADHD, but rather as a standardized measure of attention and inhibitory control—dimensions highly relevant to impulsivity and dopaminergic function [17,19,20].

To reduce potential confounding effects of sex-specific hormonal modulation on dopaminergic activity and impulsivity, we limited our sample to male participants. This approach aligns with prior genetic studies highlighting sex differences in dopaminergic signaling pathways and ensures greater interpretability of the genotype–phenotype associations [11,21,22].

The present study examined whether the *DRD2* rs1076560 polymorphism influences impulsivity and related cognitive–behavioral traits in stimulant-dependent and non-dependent men. We tested genotype-by-group (stimulant-dependent vs. control) interactions across validated psychological scales, hypothesizing that rs1076560 would predict differences in attentional, motor, and non-planning impulsivity, self-reported attentional difficulties, and hedonic capacity. Clarifying these gene–trait associations may enhance our understanding of addiction vulnerability and support the development of genetically informed strategies for stimulant use disorders [10,23,24].

## 2. Materials and Methods

### 2.1. Materials

The study sample comprised 517 male participants, including 235 individuals diagnosed with stimulant dependence and 282 non-addicted controls. The study protocol was approved by the Bioethics Committee of the Pomeranian Medical University in Szczecin (approval no. KB-0012/106/16 on 17 October 2016), and written informed consent was obtained from all participants prior to inclusion. Data collection took place at the Independent Laboratory of Behavioral Genetics and Epigenetics, Pomeranian Medical University in Szczecin. Both groups were recruited between 13 March 2016 and 16 April 2019. Participants in the stimulant dependence group were recruited from addiction treatment facilities, whereas control subjects were volunteers with no history of substance use disorders, as confirmed by a structured psychiatric interview (Mini International Neuropsychiatric Interview, MINI) conducted by a certified psychiatrist. In addition, all participants completed the Barratt Impulsiveness Scale—Version 11 (BIS-11), the Adult ADHD Self-Report Scale (ASRS v1.1), and the Snaith–Hamilton Pleasure Scale (SHAPS). Exclusion criteria for both groups included intellectual disability, dementia, developmental disorders, history of traumatic brain injury, current suicidal risk, and significant somatic illnesses (e.g., cardiovascular, endocrine, neurological, or metabolic diseases) that could affect cognitive functioning or mental health. The study was observational and aimed to examine the interaction between personality-related traits and the *DRD2* rs1076560 polymorphism in individuals with and without stimulant dependence.

**Figure 1:**
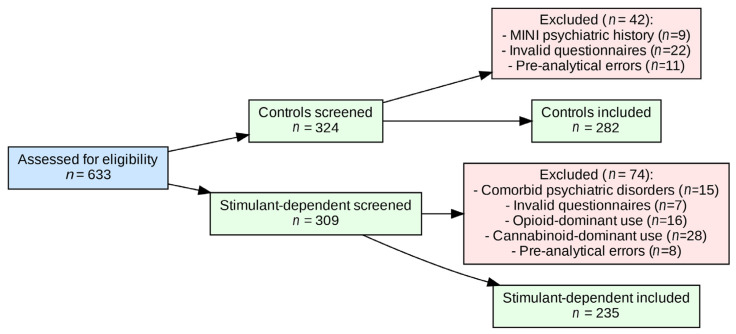
Flow diagram of participant recruitment, exclusions, and final inclusion.

A total of 633 men were assessed for eligibility. After exclusions, 282 controls and 235 stimulant-dependent participants were included in the analyses. The detailed recruitment cascade is shown in Figure 1.

### 2.2. Measures

To assess the psychological traits of interest, participants completed three self-report questionnaires measuring impulsivity, attention-related difficulties, and hedonic capacity.

Barratt Impulsiveness Scale Version 11 (BIS-11):

Impulsivity was assessed using the BIS-11, a 30-item self-report questionnaire designed to evaluate three dimensions of impulsivity: Attentional Impulsivity (AI), Motor Impulsivity (MI), and Non-Planning Impulsivity (NI). Each item is rated on a 4-point Likert scale (from rarely/never to almost always/always), with higher scores indicating greater impulsivity. The BIS-11 has been validated across a range of clinical and non-clinical populations and is widely used in personality and addiction research.

Adult ADHD Self-Report Scale (ASRS v1.1):

ADHD-related symptoms were measured using the 18-item Adult ADHD Self-Report Scale (ASRS v1.1), developed by the World Health Organization. The scale captures the core symptom dimensions of inattention and hyperactivity/impulsivity based on DSM-IV criteria. Responses are given on a 5-point Likert scale, ranging from 0 (never) to 4 (very often). The ASRS v1.1 is a well-established screening tool with strong psychometric properties in adult populations.

Snaith–Hamilton Pleasure Scale (SHAPS):

Hedonic capacity was assessed using the Snaith–Hamilton Pleasure Scale (SHAPS), a 14-item self-report instrument designed to measure the ability to experience pleasure in everyday activities. Respondents indicate their level of agreement with statements about pleasurable experiences in various domains (e.g., social, sensory, interests, and hobbies), using a 4-point scale. The SHAPS is a widely validated tool for detecting anhedonia, particularly in clinical populations with mood or affective disorders (see Table 1).

### 2.3. Genotyping

Genomic DNA was obtained from whole venous blood samples. DNA was isolated according to standard phenol–chloroform procedures, following established laboratory protocols (Roche Diagnostics, Mannheim, Germany). The concentration and purity of the extracted DNA were verified spectrophotometrically and all samples were stored at −20 °C until analysis.

Genotyping of the *DRD2* rs1076560 (A > C) polymorphism was performed using the real-time PCR method with fluorescence resonance energy transfer (FRET) probes on a LightCycler^®^ 480 II System (Roche Diagnostics, Basel, Switzerland), following the manufacturer’s protocol. The fluorescence signal was continuously monitored during the melting phase and plotted against temperature to generate melting curves for each sample. Characteristic melting peaks were observed at approximately 57.36 °C for the A allele and 64.40 °C for the C allele, enabling genotype determination. Each run included both positive and negative controls.

### 2.4. Statistical Analysis

The distribution of *DRD2* rs1076560 genotypes was assessed for Hardy–Weinberg equilibrium (HWE) using an online calculator (GeneRiskCalc: https://sites.google.com/view/GeneRiskCalc/home?authuser=0, accessed on 29 September 2025). Differences in genotype frequencies between individuals with stimulant dependence and healthy controls were tested using the chi-square (χ^2^) test.

Scores from the Barratt Impulsiveness Scale (BIS-11; total and subscales), Adult ADHD Self-Report Scale (ASRS v1.1; total and subscales), and Snaith–Hamilton Pleasure Scale (SHAPS) were compared between groups using the Mann–Whitney U test. The same test was used to compare these scores between genotype subgroups within each study group. In addition, group (stimulant-dependent vs. control) × genotype (rs1076560) effects on BIS-11, ASRS, and SHAPS scores were analyzed using a factorial ANOVA with 2 factors. All statistical analyses were performed using STATISTICA version 13 (TIBCO Software Inc., Palo Alto, CA, USA) and JASP 0.95.2 (University of Amsterdam) for Windows (Microsoft Corporation, Redmond, WA, USA). A two-tailed *p*-value < 0.05 was considered statistically significant. Statistical reporting was aligned with the SAMPL (Statistical Analyses and Methods in the Published Literature) guidelines, with *p*-values, test statistics, and effect sizes reported consistently to improve clarity and reproducibility. The study was exploratory in nature; therefore, no a priori sample size calculation was performed. The sample size was determined by the availability of eligible participants during the recruitment period. All analyses were conducted on complete cases. Participants with missing or invalid questionnaire responses or genotyping failures were excluded prior to analysis, as detailed in the flow diagram (Figure 1).

## 3. Results

**Figure 2:**
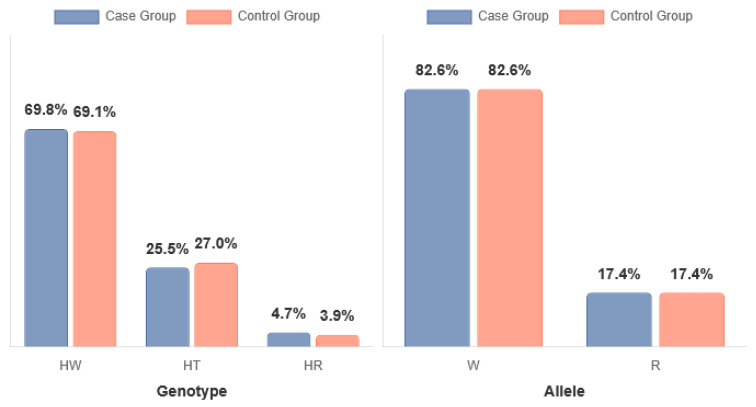
*DRD2* rs1076560 genotypes in stimulant-dependent (SD; case group) and control groups. HW = C/C (wild type); HT = A/C (heterozygote); HR = A/A (recessive); W = C allele; R = A allele.

The genotype frequency distribution in the stimulant-dependent group deviated from the Hardy–Weinberg equilibrium (HWE), whereas the control group was in full compliance with HWE. The observed deviation in the stimulant-dependent group likely reflects the selective nature of this cohort and the moderate sample size, rather than genotyping errors (see Table 2 and Table 3, Figure 2).

No statistically significant differences were observed in the distribution of *DRD2* rs1076560 genotypes between individuals with stimulant dependence and healthy controls. The proportion of genotypes was comparable in both groups: C/C (0.70 vs. 0.69), A/C (0.26 vs. 0.27), and A/A (0.05 vs. 0.04), with the chi-square test yielding χ^2^ = 0.2890, *p* = 0.8655. Similarly, there were no significant differences in allele frequencies between the groups. The frequency of the C allele was identical in both the stimulant-dependent and control participants (0.83 vs. 0.83), as was the A allele (0.17 vs. 0.17), χ^2^ = 0.0009, *p* = 0.9761 (see Table 4).

For reference, genotype and allele frequencies observed in our sample were compared with those reported in previous Poland-derived or neighboring population studies. The results of these comparisons are provided in Supplementary Figure S3.

Marital status and education of the stimulant-dependent group are reported in Table 5. Most participants were single and had secondary or lower education. The mean age of onset of stimulant dependence was 15.99 years (SD = 3.28), and the mean illness duration was 11.51 years (SD = 6.04).

**Figure 3:**
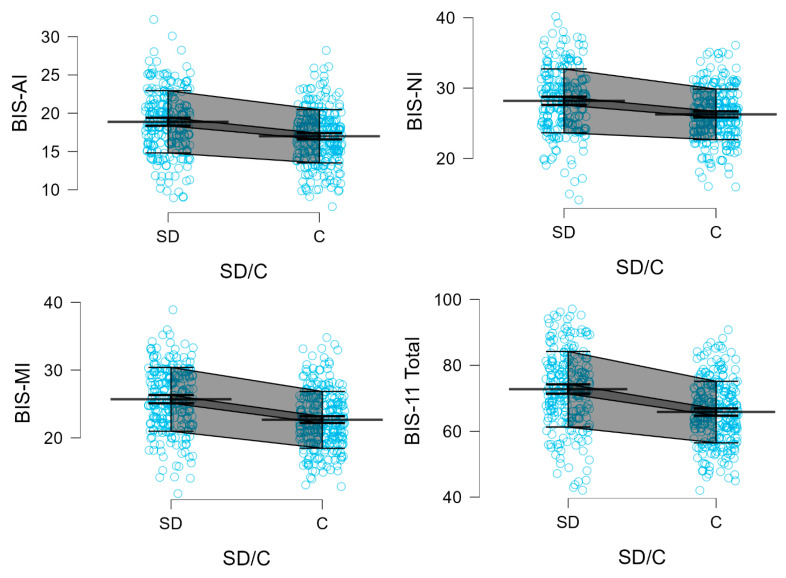

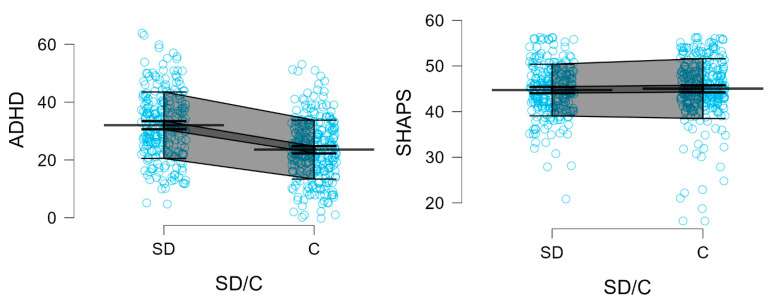
BIS-11 subscales, BIS-11 total, ADHD, and SHAPS scores in stimulant-dependent (SD) and control (C) groups. Each blue dot represents an individual participant’s score. Mean (single bold line), SD error (gray field), and 95% CI (dark gray field) are shown.

Figure 3 presents the Mean, SD, 95% CI for BIS, ADHD, and SHAPS scores in stimulant-dependent and control groups.

Descriptive statistics for the BIS-11 scores, including means and standard deviations for all subscales and the total score, are presented separately for the stimulant dependence group and the control group in Table 5.

Participants in the stimulant dependence group scored significantly higher than controls on all subscales and the total score of the BIS-11, as well as on the ADHD trait scale. Specifically, elevated scores were observed for Attentional Impulsivity (AI) (18.88 vs. 16.99; *Z* = 5.469; *p* < 0.0001), Motor Impulsivity (MI) (25.69 vs. 22.65; *Z* = 7.480; *p* < 0.0001), and Non-Planning Impulsivity (NI) (28.17 vs. 26.25; *Z* = 5.166; *p* < 0.0001). The BIS-11 total score was also significantly higher among stimulant users (72.73 vs. 65.82; *Z* = 7.259; *p* < 0.0001), as was the score on the ADHD Self-Report Scale (32.01 vs. 23.58; *Z* = 8.088; *p* < 0.0001).

In contrast, no significant difference between groups was observed in scores on the Snaith-Hamilton Pleasure Scale (SHAPS) (44.73 vs. 45.04; *Z* = –1.255; *p* = 0.2093). Detailed descriptive and inferential statistics for all comparisons are presented in Table 5, and results of the two-way factorial ANOVA are summarized in Table 6. Furthermore, two variables, referred to as Factor 1 and Factor 2, were generated using Principal Component Analysis (PCA) and subjected to ANOVA. Main Component 1 clustered the BIS-AI, BIS-MI, BIS-NI, BIS-11 Total, and ADHD scores, whereas the SHAPS score constituted Main Component 2 (Table 7; Supplementary Table S1; Supplementary Figures S1 and S2).

BIS—Attentional Impulsivity (AI)

**Figure 4:**
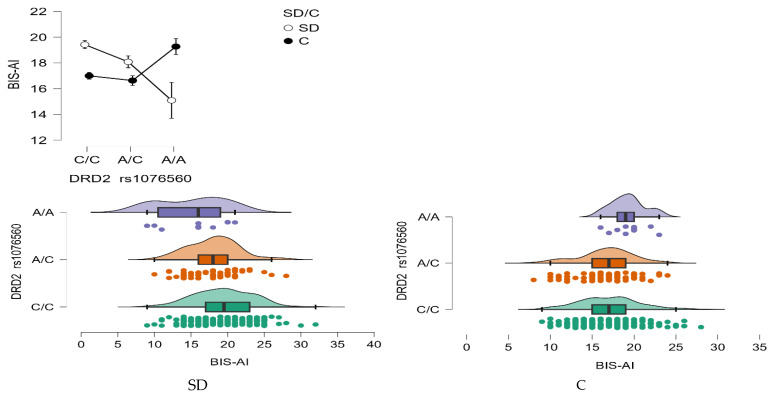
Interaction between group (stimulant-dependent vs. control) and *DRD2* rs1076560 genotype on BIS-AI scores.

A statistically significant main effect of the *DRD2* rs1076560 genotype was observed for Attentional Impulsivity (BIS-AI) scores (F_2,509_ = 3.08, *p* = 0.0469, η^2^ = 0.012; Figure 4). The observed statistical power for this effect was 59%, with approximately 1% of the variance in BIS-AI scores explained by differences in genotype.

Importantly, there was also a significant interaction between genotype and group status (stimulant dependence vs. control) on BIS-AI scores (F_2,509_ = 8.57, *p* = 0.0002, η^2^ = 0.033), with a statistical power of 97%. This interaction accounted for approximately 3% of the variance, suggesting that the impact of the *DRD2* rs1076560 polymorphism on attentional impulsivity may differ depending on addiction status (Figure 4).

Detailed results from the post hoc comparisons are provided in Table 8. Individuals with stimulant dependence carrying the C/C genotype exhibited significantly higher BIS-AI scores compared to those with the A/C and A/A genotypes, as well as compared to control participants with the C/C and A/C genotypes. Furthermore, stimulant-dependent individuals with the A/C genotype scored significantly higher than those with the A/A genotype, and also higher than control subjects with C/C and A/C genotypes.

BIS—Motor Impulsivity (MI)

**Figure 5:**
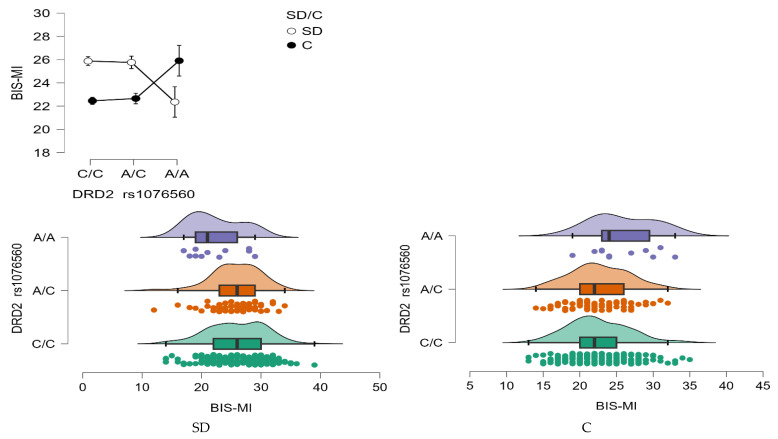
Interaction between group (stimulant-dependent vs. control) and *DRD2* rs1076560 genotype on BIS-MI scores.

A significant interaction effect was found between *DRD2* rs1076560 genotype and group status (stimulant dependence vs. control) on Motor Impulsivity (BIS-MI) scores (F_2,509_ = 6.52, *p* = 0.0016, η^2^ = 0.025; Figure 5). The effect size indicated that approximately 2.5% of the variance in BIS-MI scores could be attributed to the genotype × group interaction, with a statistical power of 91%.

Post hoc comparisons (Table 8) revealed that individuals with stimulant dependence carrying either the C/C or A/C genotype scored significantly higher on the BIS-MI scale than those with the A/A genotype within the same clinical group, as well as higher than control subjects with C/C or A/C genotypes. Additionally, control group participants with the A/A genotype demonstrated significantly higher BIS-MI scores compared to their peers with C/C and A/C genotypes, suggesting that the A/A variant may be associated with increased motor impulsivity regardless of addiction status.

BIS—Non-Planning Impulsivity (BIS-NI)

**Figure 6:**
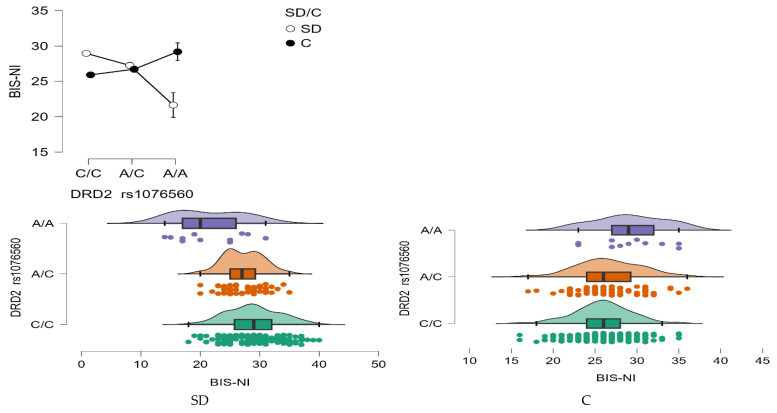
Interaction between group (stimulant-dependent vs. control) and *DRD2* rs1076560 genotype on BIS-NI scores.

A statistically significant main effect of group was found for BIS-NI scores, with individuals in the stimulant dependence group scoring higher than controls (F_1,509_ = 4.68, *p* = 0.031, η^2^ = 0.009; Figure 6). The statistical power for this effect was 58%, accounting for approximately 1% of the variance.

In addition, a significant main effect of *DRD2* rs1076560 genotype was observed (F_2,509_ = 3.17, *p* = 0.0427, η^2^ = 0.012), with a power of 61%, also explaining around 1% of the variability in BIS-NI scores. Crucially, the interaction between genotype and group status was highly significant (F_2,509_ = 22.14, *p* < 0.0001, η^2^ = 0.080), with a statistical power of 99%. This interaction accounted for approximately 8% of the variance in non-planning impulsivity scores, suggesting a robust modulatory effect of genotype depending on addiction status (Figure 6).

As detailed in Table 8, stimulant-dependent participants with the C/C genotype scored significantly higher on the BIS-NI scale compared to both those with A/C and A/A genotypes, as well as compared to control individuals with C/C and A/C genotypes. Within the stimulant group, individuals with the A/C genotype also demonstrated elevated BIS-NI scores relative to those with the A/A genotype, and to control participants with C/C and A/C genotypes.

BIS-11 Total Score

**Figure 7:**
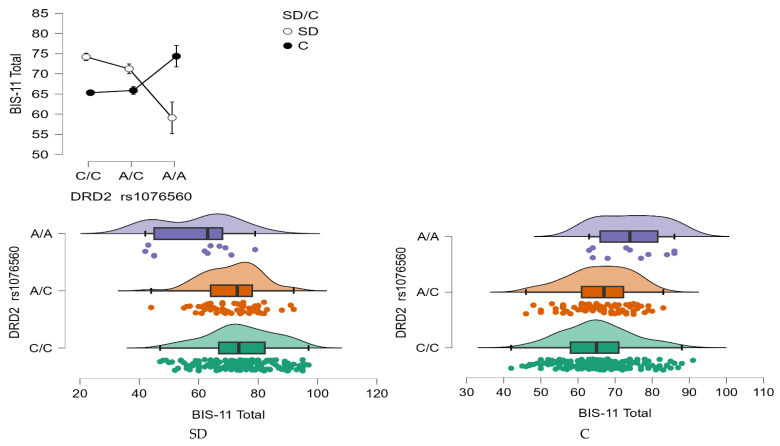
Interaction between group (stimulant-dependent vs. control) and *DRD2* rs1076560 genotype on BIS-11 Total scores.

A statistically significant interaction effect was found between *DRD2* rs1076560 genotype and group status (stimulant dependence vs. control) on the BIS-11 total score (F_2,509_ = 15.44, *p* < 0.0001, η^2^ = 0.057; Figure 7). The observed power for this interaction was 99%, and approximately 6% of the variance in total impulsivity scores could be explained by the interaction between genotype and addiction status.

As shown in Table 8, individuals with stimulant dependence who carried either the C/C or A/C genotype scored significantly higher on the BIS-11 total scale compared to those with the A/A genotype and to control participants with C/C and A/C genotypes. Additionally, control group participants with the A/A genotype exhibited significantly higher total BIS-11 scores than both stimulant-dependent individuals with the same genotype and controls with the C/C genotype.

ADHD scale

A significant main effect of group status was observed for the ADHD trait scale, with individuals in the stimulant dependence group scoring higher than controls (F_1,509_ = 7.43, *p* = 0.0066, η^2^ = 0.014). The effect size indicated that approximately 1% of the variance in ADHD scores could be attributed to group differences, with an observed power of 78%.

**Figure 8:**
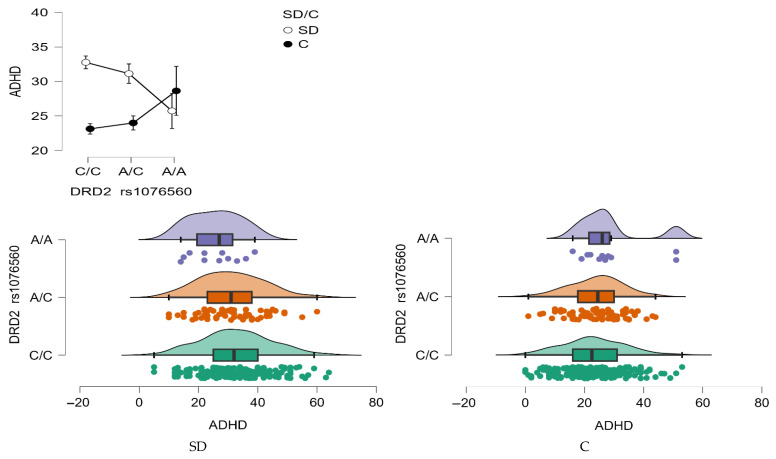
Interaction between group (stimulant-dependent vs. control) and *DRD2* rs1076560 genotype on ADHD scores.

Moreover, a significant interaction effect between *DRD2* rs1076560 genotype and group status was found (F_2,509_ = 3.83, *p* = 0.0222, η^2^ = 0.015; Figure 8), with a power of 69%. This interaction explained approximately 1.5% of the variance in ADHD trait scores, suggesting a modest but meaningful role of genotype in modulating attentional traits based on addiction status.

As summarized in Table 8, stimulant-dependent individuals with the C/C genotype had significantly higher ADHD scores compared to those with the A/A genotype, as well as compared to control subjects with C/C and A/C genotypes. Additionally, stimulant-dependent individuals carrying the A/C genotype also scored significantly higher than controls with C/C and A/C genotypes.

Snaith-Hamilton Pleasure Scale (SHAPS)

No significant group differences were observed on the SHAPS scores, with stimulant-dependent and control participants reporting comparable levels of hedonic tone (Table 5). Similarly, the 2 × 3 factorial ANOVA did not reveal any significant effects of the *DRD2* rs1076560 genotype, nor any interaction between genotype and group status (Table 6). These findings suggest that neither stimulant dependence nor the rs1076560 variant was associated with altered pleasure capacity in the present sample.

Model PCA Main components 1

**Figure 9:**
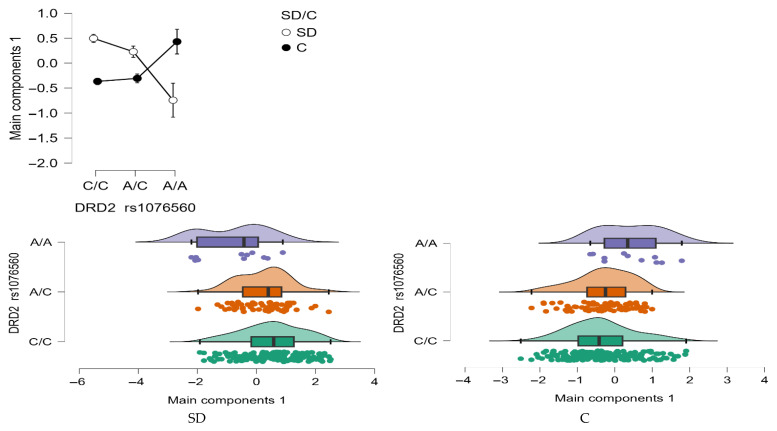
Interaction between group (stimulant-dependent vs. control) and *DRD2* rs1076560 genotype on PCA Main components 1.

A statistically significant interaction effect was found between *DRD2* rs1076560 genotype and group status (stimulant dependence vs. control) on the Model PCA Main components 1 (F_2,509_ = 13.30, *p* < 0.0001, η^2^ = 0.051; Figure 9). The observed power for this interaction was 99%, and approximately 5% of the variance in total impulsivity scores could be explained by the interaction between genotype and addiction status.

As shown in Table 8, individuals with stimulant dependence who carried either the C/C or A/C genotype scored significantly higher on the Model PCA Main components 1compared to those with the A/A genotype and to control participants with C/C and A/C genotypes. Additionally, control group participants with the A/A genotype exhibited significantly higher total Model PCA Main components 1 than both stimulant-dependent individuals with the same genotype and controls with the C/C and A/C genotype.

**Figure 10:**
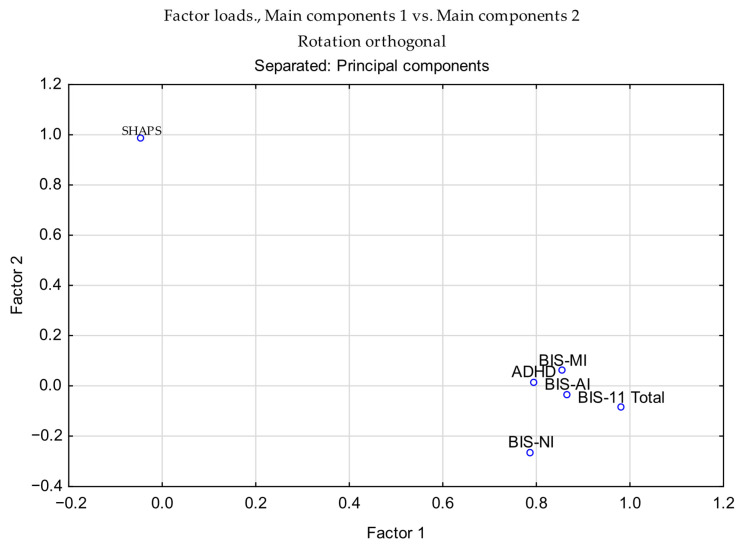
Loading plot of psychometric variables on the first two principal components (orthogonal rotation). BIS-11 subscales (AI, MI, NI, and Total) and ADHD cluster on Component 1, while SHAPS loads uniquely on Component 2.

The PCA results indicated that BIS-11 subscales (AI, MI, NI, and Total) and ADHD scores loaded strongly on the first component, whereas SHAPS scores were uniquely associated with the second component (Table 7). This pattern suggests that impulsivity-related measures cluster together, while anhedonia is captured as a distinct factor. Detailed coefficients of psychometric variables on the first two principal components are provided in the Supplementary Materials (Supplementary Table S1), together with the correlation matrix of BIS-11, ADHD, and SHAPS scores (Supplementary Table S2). To visualize these relationships, Figure 10 presents the loading plot of psychometric variables on the first two principal components, highlighting the clustering of impulsivity-related scores along PC1 and the distinct loading of SHAPS on PC2.

To illustrate the relationships among variables, Figure 10 presents the loading plot of psychometric measures on the first two principal components. This visualization highlights the clustering of impulsivity-related scores along PC1 and the distinct loading of SHAPS on PC2. Additional PCA-related visualizations remain available in the Supplementary Materials, including the scree plot of eigenvalues (Supplementary Figure S1) and pairwise scatterplots with correlation matrices for BIS-11 subscales, ADHD, and SHAPS scores (Supplementary Figure S2). Collectively, these results illustrate the variance explained by successive components, highlight variable associations with PC1 and PC2, and confirm the absence of critical outliers in the dataset.

## 4. Discussion

### 4.1. Clinical Contexts—Addictions

The present findings add to evidence implicating the dopaminergic system in addictive disorders characterized by impaired inhibitory control [25,26,27]. In this sample of adult men, stimulant-dependent individuals exhibited significantly higher levels of trait impulsivity (BIS-11 total and subscales: η^2^ = 0.057–0.080) and attentional dysregulation (ASRS: η^2^ = 0.015) compared to controls. These results are consistent with previous studies linking *DRD2*-related alterations to substance use disorders. In particular, reduced D2 receptor availability in striatal and prefrontal regions has been associated with poorer response inhibition, heightened reward sensitivity, and steeper delay discounting [28,29].

Significant interaction effects between stimulant dependence status and *DRD2* rs1076560 genotype were observed for the BIS-11 total score, all BIS-11 subscales, and the ASRS total score. Post hoc analyses indicated that in the stimulant-dependent group, C/C homozygotes scored significantly higher on BIS-11 Attentional Impulsivity compared with both A/C controls (*p* < 0.0001) and C/C controls (*p* < 0.0001). A similar pattern for C/C stimulant-dependent individuals was observed for the ASRS total score. However, in the control group, A/A homozygotes showed higher scores on Motor Impulsivity, Non-Planning Impulsivity, and the BIS-11 total score compared with C/C controls. These findings indicate that the effect of rs1076560 on impulsivity-related traits is context-dependent. In stimulant-dependent individuals, the C/C genotype confers higher attentional dysregulation, while in non-dependent men A/A genotype may be associated with elevated impulsivity. This pattern supports the view that the *DRD2* rs1076560 polymorphism influences vulnerability to stimulant use disorders by modulating neurofunctional pathways related to cognitive control [9,24,30]. Prior imaging studies have reported that A allele carriers show altered activation in frontal–striatal circuits, with more efficient top-down regulation during cognitive tasks [7,31]. The present results suggest that such mechanisms may be less effective in C/C homozygotes in the context of stimulant exposure. In contrast, in non-dependent individuals, the A/A genotype may predispose to higher impulsivity on certain dimensions.

No significant main effects or genotype-by-group interactions were found for SHAPS scores, indicating no detectable differences in hedonic capacity across genotypes or diagnostic categories. This may reflect limitations in the sensitivity of the SHAPS to detect subtle anhedonic features in non-depressed populations or among individuals in early or variable phases of stimulant use [32,33,34,35]. Dynamic behavioral measures, such as probabilistic reward learning or effort-based decision-making, may offer greater sensitivity for detecting mild impairments in reward responsiveness [36,37]. It is also possible that hedonic tone in stimulant users is shaped more by other neurochemical systems, such as serotonergic or endogenous opioid pathways [3]. While *DRD2* variation (e.g., Taq1A) has been linked to affective reward processing, rs1076560 may exert more specific effects on cognitive–behavioral domains, such as impulsivity and executive control, rather than on hedonic responses [7].

### 4.2. Non-Clinical Contexts (Sport and Personality Traits)

Although the present sample did not include athletes, previous research suggests that the implications of rs1076560 extend to non-clinical populations. This polymorphism has been associated with individual differences in traits such as novelty seeking, decision-making, and executive function [38,39]. In healthy cohorts, A allele carriers have demonstrated better performance on tasks requiring sustained attention and impulse regulation, potentially related to altered D2S/D2L receptor expression that favors postsynaptic modulation [7,31,40].

This variant may also contribute to optimizing behavioral flexibility and cognitive control in high-demand environments such as competitive sports, military service, or leadership roles—contexts in which attentional regulation and inhibitory control are critical [41]. The present findings complement this literature by indicating that the behavioral profile associated with the A allele may not be uniformly protective. Instead, it appears to be influenced by environmental context, with stimulant dependence disrupting the potential advantages observed in non-clinical settings [42,43].

Our findings may contribute to the development of genetically informed strategies for stimulant use disorders [44]. Although this study cannot establish causal relationships, the observed association between *DRD2* rs1076560 polymorphism, impulsivity, and stimulant dependence suggests that integrating genetic information with behavioral and clinical assessment could eventually support personalized prevention and intervention approaches. Such strategies, though still at an early stage, might help identify high-risk individuals and guide tailored therapeutic options [45].

### 4.3. Translational Implications and Future Directions

The observed genotype-by-group interactions highlight potential differences in vulnerability that could eventually inform targeted prevention or intervention approaches [7,9,42]. For example, individuals with the C/C genotype may be more prone to stimulant-related cognitive dysregulation and might benefit from early identification and interventions aimed at strengthening executive function [24].

At present, however, clinical application remains premature. Replication in larger and more diverse samples, as well as longitudinal designs, will be essential to determine whether *DRD2* genotypes predict substance use trajectories or treatment responsiveness [9,46]. Integrating genetic data with behavioral measures, neuroimaging, and environmental information could support the development of multidimensional risk profiles to guide intervention planning [27,42]. Beyond impulsivity, future research should also investigate motivational, affective, and social–cognitive dimensions linked to *DRD2* variation to advance comprehensive neurogenetic models of addiction [7].

Future studies employing behavioral or neurobiological measures of reward processing may clarify the relationship between *DRD2* rs1076560 variation and hedonic capacity. In addition, our results highlight the potential relevance of *DRD2* rs1076560 genotyping in guiding future research on addiction vulnerability. While the findings are promising, clinical translation will require replication in larger and more diverse samples, as well as integration with multimodal assessment strategies, before genotype-informed interventions can be reliably implemented.

### 4.4. Limitations

Several limitations must be acknowledged. First, the exclusive inclusion of male participants limits generalizability. Dopaminergic signaling and impulsivity profiles differ by sex, and future studies should assess whether the observed effects replicate in female samples [47,48]. Second, reliance on self-report tools (BIS-11, ASRS, SHAPS) restricts our ability to capture real-time cognitive dynamics. Integrating behavioral paradigms and neuroimaging data would strengthen causal inferences [49]. Third, although our sample was sufficient for detecting moderate effects, larger and more demographically diverse cohorts are needed to confirm the robustness and specificity of the observed gene–environment interactions [50,51]. Fourth, stimulant use severity and duration were not controlled for, which may confound the relationship between impulsivity and genotype. Fifth, there was a significant age difference between the stimulant-dependent and control groups (mean difference ≈ 5 years). Such age-related changes in personality traits, impulsivity, and reward sensitivity may partially account for the observed differences. This should therefore be considered a potential confounder. We did not include age as a covariate in the factorial ANOVA models; therefore, the possibility of residual confounding by age cannot be excluded. Finally, we focused solely on rs1076560; future research should examine additional *DRD2* variants and gene–gene interactions (e.g., with *DAT1*, *COMT*) to better characterize polygenic influences. In addition, as this was an exploratory study, no a priori sample size calculation was performed. The sample size was determined by the availability of eligible participants, which may limit the statistical power to detect smaller effects. Moreover, only a limited number of studies from Poland or neighboring countries report complete genotype distribution data for rs1076560, which restricts the scope of regional comparisons [23,52].

## 5. Conclusions

This study provides novel evidence that the *DRD2* rs1076560 polymorphism interacts with stimulant dependence status to influence impulsivity-related traits in adult men. Significant genotype-by-group interactions emerged across all BIS-11 subscales and for ASRS scores. In the stimulant-dependent group, the highest levels of attentional impulsivity and attentional dysregulation were observed among C/C homozygotes, whereas in the control group, A/A homozygotes showed higher motor and non-planning impulsivity, as well as higher BIS-11 total scores, compared with C/C controls. These findings support a role of the *DRD2* rs1076560 variant in shaping behavioral vulnerabilities associated with stimulant use, with effects that differ depending on the presence or absence of dependence.

Although no effects were found for hedonic capacity as measured by the SHAPS, this may reflect the scale’s limited sensitivity in non-clinical or subclinical populations. Our results underscore the importance of considering gene–environment interactions in models of addiction risk.

## Figures and Tables

**Table 1:** Age, BIS-11, ADHD, and SHAPS scores in stimulant-dependent and control groups.

AGE, BIS-11, ADHD, SHAPS	SD (*n*= 235) M ± SD	Control (*n* = 282) M ± SD	Z	(*p*-Value)
AGE (years)	27.43 ± 5.72	22.08 ± 4.14		
BIS-AI	18.88 ± 4.07	16.99 ± 3.49	5.469	<0.0001 *
BIS-MI	25.69 ± 4.70	22.65 ± 4.18	7.480	<0.0001 *
BIS-NI	28.17 ± 4.54	26.25 ± 3.60	5.166	<0.0001 *
BIS-11 Total	72.73 ± 11.46	65.82 ± 9.32	7.259	<0.0001 *
ADHD	32.01 ± 11.51	23.58 ± 10.19	8.088	<0.0001 *
SHAPS	44.73 ± 5.65	45.04 ± 6.56	−1.255	0.2093

*p*—statistical significance (Mann–Whitney U test); n—number of subjects; M ± SD—mean ± standard deviation; *—statistically significant differences; SD- Stimulant-dependent;.

**Table 2:** Hardy–Weinberg equilibrium analysis for the *DRD2* rs1076560 genotypes in stimulant-dependent (SD) and control groups.

	Genotypes	Observed (Expected)	Allele Freq	χ^2^ (*p* Value)
SD *n* = 235	C/C	164/69.8% (160.2)	W (Wild Allele): 82.6 R (Recessive Allele): 17.4	3.036 (0.0815)
	A/C	60/25.5% (67.6)		
	A/A	11/4.7% (7.2)		
Control *n* = 282	C/C	195/69.1% (192.5)	W (Wild Allele): 82.6 R (Recessive Allele): 17.4	1.063 (0.3025)
	A/C	76/27.0% (81.0)		
	A/A	11/3.9% (8.5)		

*n*—number of subjects; *p*—statistical significance χ^2^ test; SD—Stimulant-dependent.

**Table 3:** Association analysis results.

Genetic Model	Comparison	Odds Ratio (95% CI)	Z-Score	*p*-Value	Adj. Significance
Recessive	HR vs. (HT + HW)	1.21 (0.51–2.84)	0.4370	0.6621 1.0000	N S
Dominant	(HR + HT) vs. HW	0.97 (0.67–1.41)	−0.1569	0.8753 1.0000	N S
Overdominant	HT vs. (HR + HW)	0.93 (0.63–1.38)	−0.3647	0.7153 1.0000	N S
Allelic	R vs. W alleles	1.00 (0.73–1.39)	0.0299	0.9761 1.0000	N S
Codominant (HR vs. HW)	HR vs. HW	1.19 (0.50–2.81)	0.3941	0.6935 1.0000	N S
Codominant (HR vs. HT)	HR vs. HT	1.27 (0.51–3.12)	0.5138	0.6074 1.0000	N S
Codominant (HT vs. HW)	HT vs. HW	0.94 (0.63–1.40)	−0.3122	0.7549 1.0000	N S

Bonferroni correction applied (α = 0.05/7 = 0.00714). Odds Ratio (OR): Represents the odds of disease in exposed vs. unexposed. 95% CI: If includes 1.0, result is not significant at *p* < 0.05. Recessive Model: HR vs. (HT + HW)—Tests if having two recessive alleles increases risk. Dominant Model: (HR + HT) vs. HW—Tests if having at least one recessive allele increases risk. Overdominant Model: HT vs. (HR + HW)—Tests if heterozygotes have different risk. Allelic Model: Compares allele frequencies between groups. Codominant Models: Compare each genotype combination separately. N·S—Not significant.

**Table 4:** Genotype and allele frequencies of the *DRD2* rs1076560 polymorphism in stimulant-dependent and control subjects.

*DRD2* rs1076560					
	Genotypes			Alleles	
	C/C	A/C	A/A	C	A
	*n* (%)	*n* (%)	*n* (%)	*n* (%)	*n* (%)
SD	164	60	11	388	82
*n* = 235	(69.79%)	(25.53%)	(4.68%)	(82.55%)	(17.44%)
Control	195	76	11	466	98
*n* = 282	(69.15%)	(26.95%)	(3.90%)	(82.62%)	(17.38%)
χ^2^ (*p* value)	0.2890			0.0009	
	(0.8655)			(0.9761)	

*n*–number of subjects; *p*—statistical significance χ^2^ test; SD—Stimulant-dependent.

**Table 5:** Demographic and clinical characteristics of the stimulant-dependent group.

		*n*	%
Marital status	single	207	88.09
	married	12	5.11
	divorced	15	6.38
	cohabiting	1	0.43
Education	none	3	1.28
	primary	39	16.60
	lower secondary	94	40.00
	secondary	94	40.00
	higher	5	2.13
Relapse	relapse	107	45.53
	first time	128	54.47

*n*—number of subjects.

**Table 6:** Results of 2 × 3 factorial ANOVA for BIS-11, ADHD, and SHAPS scores by *DRD2* rs1076560 genotype and group (stimulant-dependent vs. control).

BIS, ADHD SHAPS, PCA	Group	*DRD2* rs1076560				ANOVA		
		C/C *n* = 359 M ± SD	A/C *n* = 136 M ± SD	A/A *n* = 22 M ± SD	Factor	F (*p* Value)	ɳ^2^	Power (alfa = 0.05)
BIS-AI	SD; *n* = 235	19.42 ± 4.05	18.08 ± 3.54	15.09 ± 4.59	intercept SD/control *DRD2* SD/control × *DRD2*	F_1,509_ = 3654.87 (*p* < 0.0001) * F_1,509_ = 0.03 (*p* = 0.8593) F_2,509_ = 3.08 (*p* = 0.0469) * F_2,509_ = 8.57 (*p* = 0.0002)	0.878 0.0001 0.012 0.033	1.000 0.054 0.593 0.967
	Control; *n* = 282	17.00 ± 3.59	16.63 ± 3.27	19.27 ± 2.05				
BIS-MI	SD; *n* = 235	25.88 ± 4.85	25.77 ± 4.14	22.36 ± 4.34	intercept SD/control *DRD2* SD/control × *DRD2*	F_1,509_ = 4897.70 (*p* < 0.0001) * F_1,509_ = 2.07 (*p* = 0.1510) F_2,509_ = 0.01 (*p* = 0.9943) F_2,509_ = 6.52 (*p* = 0.0016) *	0.906 0.004 0.00002 0.025	1.000 0.300 0.051 0.907
	Control; *n* = 282	22.46 ± 4.20	22.66 ± 3.96	25.91 ± 4.37				
BIS-NI	SD; *n* = 235	28.95 ± 4.44	27.25 ± 3.29	21.64 ± 5.78	intercept SD/control *DRD2* SD/control × *DRD2*	F_1,509_ = 7580.78 (*p* < 0.0001) * F_1,509_ = 4.68 (*p* = 0.031) * F_2,509_ = 3.17 (*p* = 0.0427) * F_2,509_ = 22.14 (*p* < 0.0001) *	0.937 0.009 0.012 0.080	1.000 0.579 0.607 0.999
	Control; *n* = 282	25.91 ± 3.40	26.71 ± 3.82	29.18 ± 4.17				
BIS-11 Total	SD; *n* = 235	74.19 ± 11.46	71.23 ± 9.31	59.09 ± 13.02	intercept SD/control *DRD2* SD/control × *DRD2*	F_1,509_ = 7441.47 (*p* < 0.0001) * F_1,509_ = 0.07 (*p* = 0.8285) F_2,509_ = 1.44 (*p* = 0.2370) F_2,509_ = 15.44 (*p* < 0.0001) *	0.936 0.0001 0.006 0.057	1.000 0.055 0.309 0.999
	Control; *n* = 282	65.31 ± 9.48	65.87 ± 8.46	74.36 ± 8.82				
ADHD	SD; *n* = 235	32.76 ± 11.80	31.13 ± 10.91	25.73 ± 8.46	intercept SD/control *DRD2* SD/control × *DRD2*	F_1,509_ = 1057.37 (*p* < 0.0001) * F_1,509_ = 7.43 (*p* = 0.0066) * F_2,509_ = 0.10 (*p* = 0.9024) F_2,509_ = 3.83 (*p* = 0.0222) *	0.675 0.014 0.0004 0.015	1.000 0.777 0.066 0.695
	Control; *n* = 282	23.13 ± 10.54	23.99 ± 8.92	28.63 ± 11.74				
SHAPS	SD; *n* = 235	44.31 ± 5.66	45.07 ± 5.53	49.09 ± 4.59	intercept SD/control *DRD2* SD/control × *DRD2*	F_1,509_ = 8893.56 (*p* < 0.0001) * F_1,509_ = 1.12 (*p* < 0.2904) F_2,509_ = 1.84 (*p* = 0.1604) F_2,509_ = 1.46 (*p* = 0.2328)	0.947 0.002 0.002 0.006	1.000 0.184 0.383 0.312
	Control; *n* = 282	45.04 ± 6.56	44.99 ± 6.75	45.36 ± 5.57				
PCA Main components 1	SD; *n* = 235	0.50 ± 1.03	0.23 ± 0.87	−0.74 ± 1.13	intercept SD/control *DRD2* SD/control × *DRD2*	F_1,509_ = 0.36 (*p* = 0.5513) F_1,509_ = 0.26 (*p* = 0.6077) F_2,509_ = 1.07 (*p* = 0.3432) F_2,509_ = 13.30 (*p* < 0.0001) *	0.0007 0.0005 0.004 0.051	0.091 0.081 0.238 0.998
	Control; *n* = 282	−0.37 ± 0.88	−0.31 ± 0.75	0.43 ± 0.82				
PCA Main components 2	SD; *n* = 235	−0.09 ± 0.94	0.09 ± 0.88	0.83 ± 0.86	intercept SD/control *DRD2* SD/control × *DRD2*	F_1,509_ = 3.15 (*p* = 0.0766) F_1,509_ = 3.03 (*p* = 0.0825) F_2,509_ = 2.34 (*p* = 0.0973) F_2,509_ = 2.51 (*p* = 0.0826)	0.006 0.006 0.009 0.010	0.425 0.412 0.474 0.502
	Control; *n* = 282	0.02 ± 1.03	−0.05 ± 1.01	0.04 ± 102				

*—significant result; SD—Stimulant-dependent; M ± SD—mean ± standard deviation; *n*—number of subjects; *p*—statistical significance (ANOVA test); η^2^—effect size (partial eta squared).

**Table 7:** PCA factor loadings with orthogonal rotation.

	PCA Main Components 1	PCA Main Components 2
BIS-AI	0.87	−0.04
BIS-MI	0.86	0.06
BIS-NI	0.79	−0.27
BIS-11 Total	0.98	−0.09
ADHD	0.79	0.01
SHAPS	−0.04	0.99
Output condition	3.70	1.05
Participation	0.62	0.18

**Table 8:** Post hoc analysis (LSD test) of interactions between group (stimulant-dependent vs. control) and *DRD2* rs1076560 genotype for BIS-AI, BIS-MI, BIS-NI, BIS-11 Total, and ADHD scores.

*DRD2* rs1076560 and BIS-AI						
	{1} M = 19.42	{2} M = 18.08	{3} M = 15.09	{4} M = 17.00	{5} M = 16.63	{6} M = 19.27
SD C/C {1}		0.0168 *	0.0002 *	<0.0001 *	<0.0001 *	0.8977
SD A/C {2}			0.0139 *	0.0479 *	0.0234 *	0.3270
SD A/A {3}				0.0963	0.1969	0.0082 *
Control C/C {4}					0.4620	0.0478 *
Control A/C {5}						0.0272 *
Control A/A {6}						
***DRD2* rs1076560 and BIS-MI**						
	{1} M = 25.88	{2} M = 25.77	{3} M = 22.36	{4} M = 22.46	{5} M = 22.66	{6} M = 25.91
SD C/C {1}		0.8665	0.0104 *	<0.0001 *	<0.0001 *	0.9819
SD A/C {2}			0.0185 *	<0.0001 *	<0.0001 *	0.9212
SD A/A {3}				0.9429	0.8355	0.0588
Control C/C {4}					0.7408	0.0116 *
Control A/C {5}						0.0221 *
Control A/A {6}						
***DRD2* rs1076560 and BIS-NI**						
	{1} M = 28.95	{2} M = 27.25	{3} M = 21.64	{4} M = 25.91	{5} M = 26.71	{6} M = 29.18
SD C/C {1}		0.0039 *	<0.0001 *	<0.0001 *	<0.0001 *	0.8489 *
SD A/C {2}			<0.0001 *	0.0201 *	0.4215 *	0.1300 *
SD A/A {3}				0.0004 *	<0.0001 *	<0.0001 *
Control C/C {4}					0.1295 *	0.0068 *
Control A/C {5}						0.0491 *
Control A/A {6}						
***DRD2* rs1076560 and BIS-11 Total**						
	{1} M = 74.19	{2} M = 71.23	{3} M = 59.09	{4} M = 65.31	{5} M = 65.87	{6} M = 74.36
SD C/C {1}		0.0522	<0.0001 *	<0.0001 *	<0.0001 *	0.9556
SD A/C {2}			0.0003 *	<0.0001 *	0.0021 *	0.3436
SD A/A {3}				0.0468 *	0.0374 *	0.0004 *
Control C/C {4}					0.6828	0.0039 *
Control A/C {5}						0.0092
Control A/A {6}						
***DRD2* rs1076560 and ADHD**						
	{1} M = 32.76	{2} M = 31.13	{3} M = 25.73	{4} M = 23.13	{5} M = 23.99	{6} M = 28.64
SD C/C {1}		0.3167	0.0365 *	<0.0001 *	<0.0001 *	0.2194
SD A/C {2}			0.1266	<0.0001 *	<0.0001 *	0.4801
SD A/A {3}				0.4377	0.6167	0.5268
Control C/C {4}					0.5588	0.0999
Control A/C {5}						0.1815
Control A/A {6}						

SD—Stimulant-dependent; *–significant statistical differences; M–mean.

## Data Availability

The data presented in this study are available on request from the corresponding author. The data are not publicly available due to privacy concerns.

## Supplementary Materials

The following supporting information can be downloaded at: https://www.mdpi.com/article/10.3390/neurolint17110182/s1, Figure S1: The Scree plot; Figure S2: Pairwise scatterplots and correlation matrix of BIS-11 subscales (AI, MI, NI, and Total), ADHD, and SHAPS scores; Figure S3: Forest plot comparing *DRD2* rs1076560 genotype and allele frequencies in the present study with previously published data from Poland and neighboring populations; Table S1: PCA factor coefficients; Table S2: Correlation matrix (BIS-11, ADHD, SHAPS).
