# Supplementary material for: Interaction Between DRD2 rs1076560 Genotype and Stimulant Dependence on Impulsivity and Self-Reported ADHD Traits in Men
Source: Neurol Int. 2025 Nov 5;17(11):182. doi: 10.3390/neurolint17110182 (PMC12655105; doi:10.3390/neurolint17110182)
Supplement: Supplementary file 1 [file neurolint-17-00182-s001.zip › neurolint-3858276 - Supplementary Figure S2.pdf]

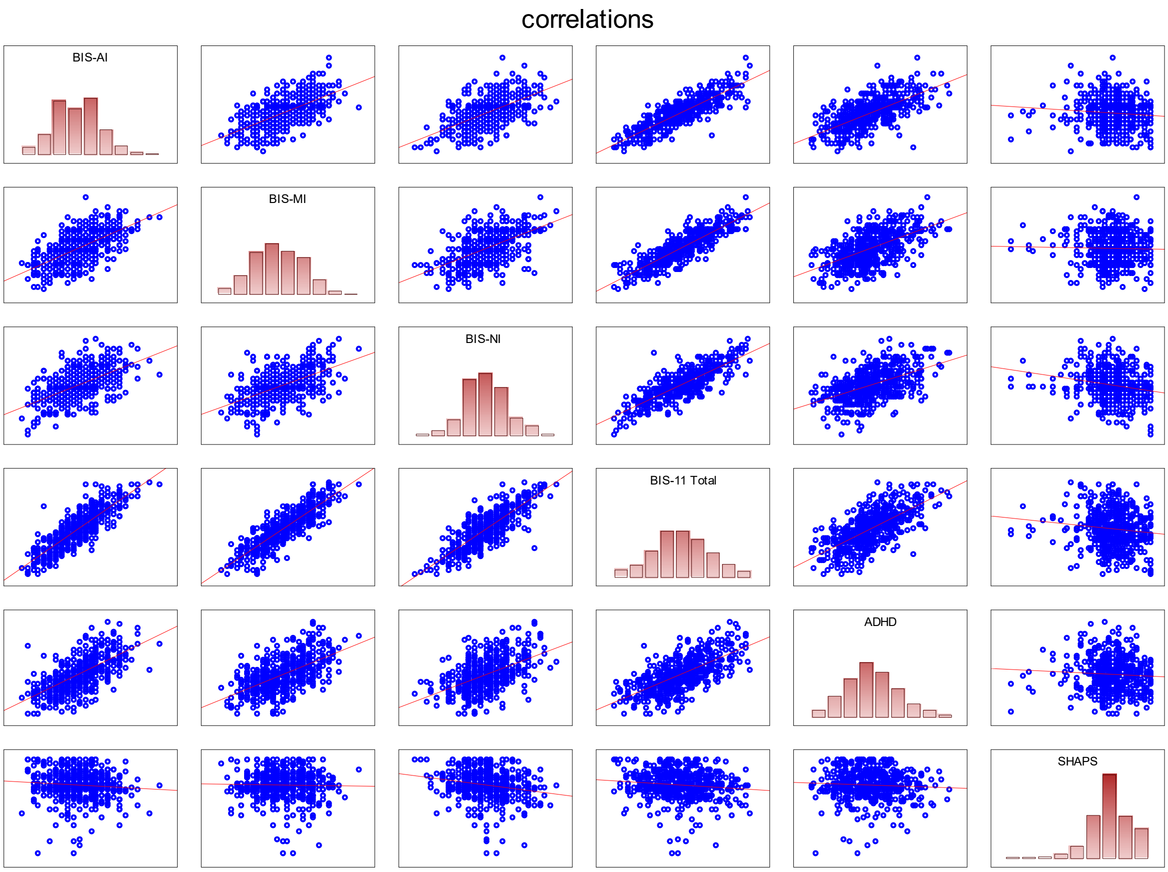

Correlations BIS-11, ADHD, and SHAPS scores.

Supplementary Figure S2. Pairwise scatterplots and correlation matrix of BIS-11 subscales (AI, MI, NI, and Total), ADHD, and SHAPS scores. Scatterplots illustrate bivariate relationships with fitted regression lines, histograms show score distributions, and correlation coefficients quantify the strength and direction of associations.
