# Supplementary material for: Interaction Between DRD2 rs1076560 Genotype and Stimulant Dependence on Impulsivity and Self-Reported ADHD Traits in Men
Source: Neurol Int. 2025 Nov 5;17(11):182. doi: 10.3390/neurolint17110182 (PMC12655105; doi:10.3390/neurolint17110182)
Supplement: Supplementary file 1 [file neurolint-17-00182-s001.zip › neurolint-3858276 - Supplementary Figure S3.pdf]

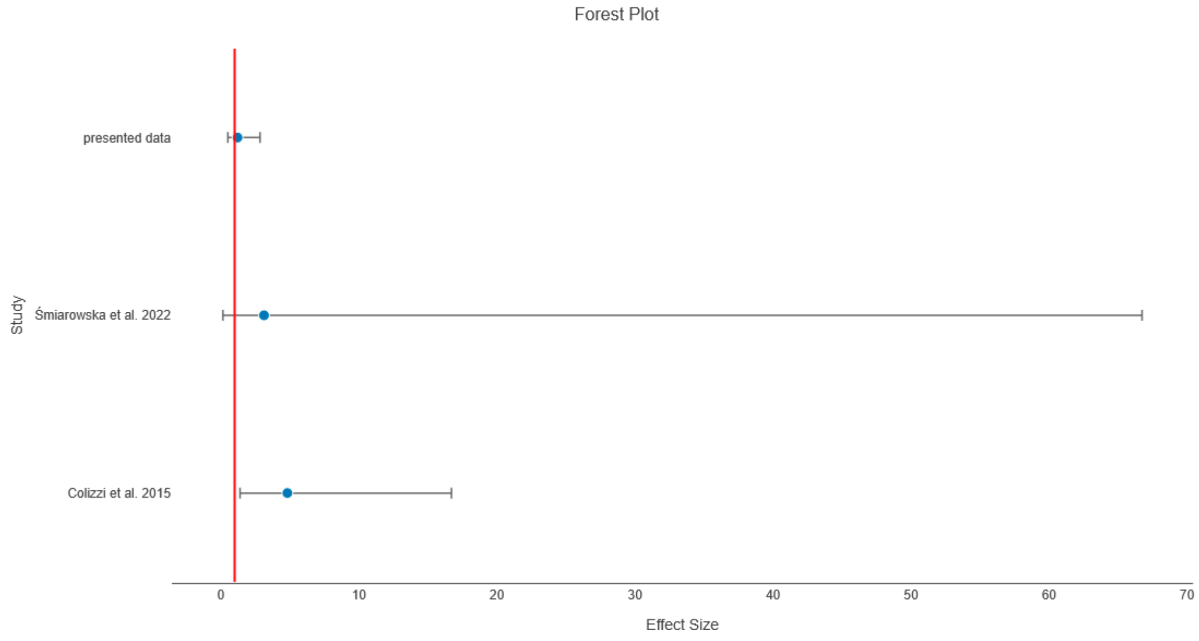

Supplementary Figure S3. Forest plot comparing *DRD2* rs1076560 genotype and allele frequencies in the present study with previously published data from Poland and neighboring populations (Śmiarowska et al., 2022; Colizzi et al., 2015).

References:

Śmiarowska, M.; Brzuchalski, B.; Grzywacz, E.; Malinowski, D.; Machoy-Mokrzyńska, A.; Pierzchlińska, A.; Białecka, M. Influence of COMT (rs4680) and DRD2 (rs1076560, rs1800497) Gene Polymorphisms on Safety and Efficacy of Methylphenidate Treatment in Children with Fetal Alcohol Spectrum Disorders. Int. J. Environ. Res. Public Health **2022**, 19, 4479. https://doi.org/10.3390/ijerph19084479.

Colizzi, M.; Iyegbe, C.; Powell, J.; Ursini, G.; Porcelli, A.; Bonvino, A.; Taurisano, P.; Romano, R.; Masellis, R.; Blasi, G.; et al. Interaction Between Functional Genetic Variation of DRD2 and Cannabis Use on Risk of Psychosis. Schizophr. Bull. **2015**, 41, 1171–1182. https://doi.org/10.1093/schbul/sbv032.
