# Supplementary material for: Interaction Between DRD2 rs1076560 Genotype and Stimulant Dependence on Impulsivity and Self-Reported ADHD Traits in Men
Source: Neurol Int. 2025 Nov 5;17(11):182. doi: 10.3390/neurolint17110182 (PMC12655105; doi:10.3390/neurolint17110182)
Supplement: Supplementary file 1 [file neurolint-17-00182-s001.zip › neurolint-3858276 - Supplementary Table S1.pdf]

Table S1. PCA factor coefficients.

	Factor coefficients – Principal components (orthogonal rotation)	
	Main components 1	Main components 2
BIS-AI	0.237	0.035
BIS-MI	0.242	0.127
BIS-NI	0.197	-0.194
BIS-11 Total	0.265	-0.006
ADHD	0.221	0.078
SHAPS.	0.069	0.955

Supplementary Table S1. Factor coefficients of psychometric variables on the first two principal components (orthogonal rotation). Loadings highlight the clustering of BIS-11 subscales and ADHD on the first component, while SHAPS loaded uniquely on the second component.
