# Supplementary material for: Interaction Between DRD2 rs1076560 Genotype and Stimulant Dependence on Impulsivity and Self-Reported ADHD Traits in Men
Source: Neurol Int. 2025 Nov 5;17(11):182. doi: 10.3390/neurolint17110182 (PMC12655105; doi:10.3390/neurolint17110182)
Supplement: Supplementary file 1 [file neurolint-17-00182-s001.zip › neurolint-3858276 - Supplementary Table S2.pdf]

Table S2. Correlation matrix (BIS-11, ADHD, SHAPS)

	Correlations					
	BIS-AI	BIS-MI	BIS-NI	BIS-11 Total	ADHD	SHAPS
BIS-AI	1.00	0.62	0.58	0.85	0.66	-0.09
BIS-MI	0.62	1.00	0.56	0.87	0.58	-0.02
BIS-NI	0.58	0.56	1.00	0.83	0.51	-0.21
BIS-11 Total	0.85	0.87	0.83	1.00	0.68	-0.12
ADHD	0.66	0.58	0.51	0.68	1.00	-0.06
SHAPS	-0.09	-0.02	-0.21	-0.12	-0.06	1.00

Supplementary Table S2. Correlation matrix of BIS-11 subscales, ADHD, and SHAPS scores. Strong positive correlations are observed among BIS-11 subscales and ADHD, whereas SHAPS scores show weak or negative associations with impulsivity-related measures.
