# Supplementary figures and images for: Interaction Between DRD2 rs1076560 Genotype and Stimulant Dependence on Impulsivity and Self-Reported ADHD Traits in Men
Source: Neurol Int. 2025 Nov 5;17(11):182. doi: 10.3390/neurolint17110182 (PMC12655105; doi:10.3390/neurolint17110182)

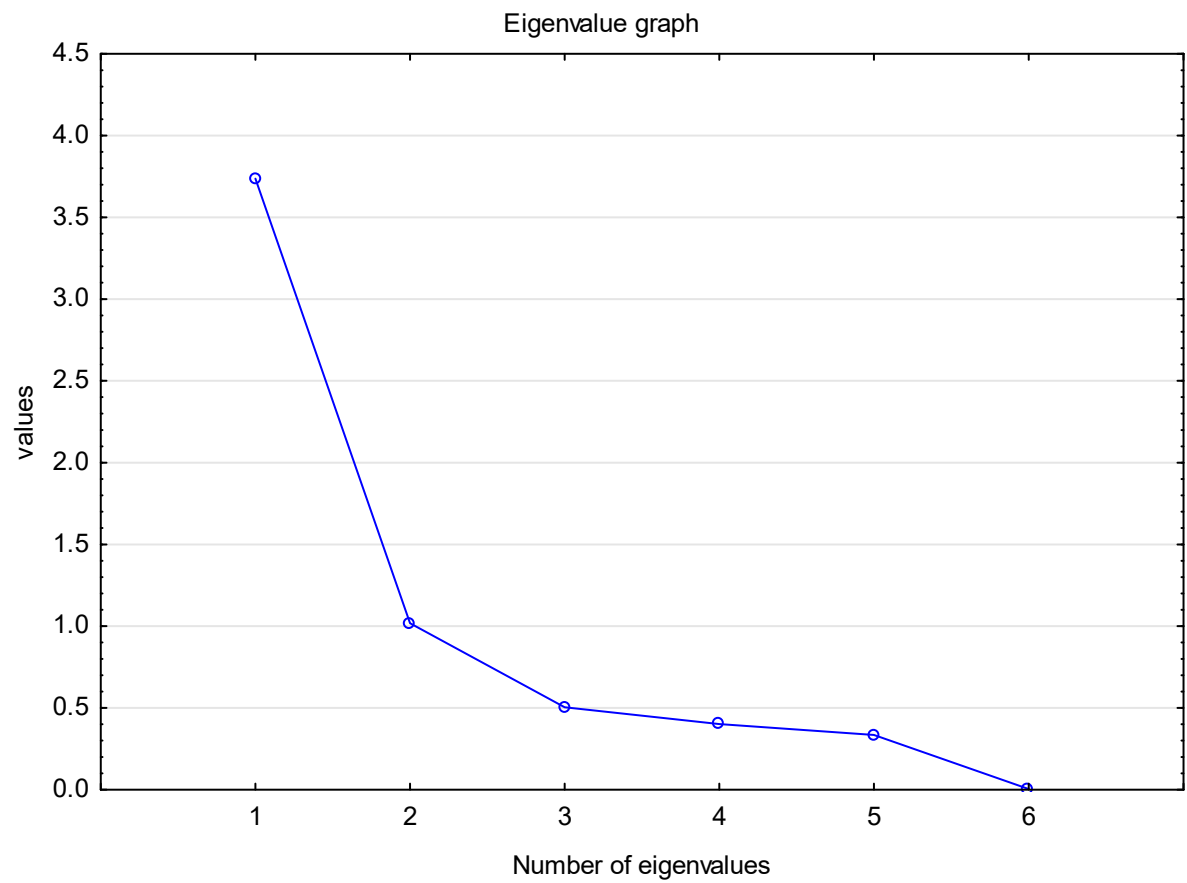

**Supplementary Figure S1. The Scree plot.**

Supplement: Supplementary file 1 [file neurolint-17-00182-s001.zip › neurolint-3858276 - Supplementary Figure S1.pdf]
